# Multistep-Ahead Air Passengers Traffic Prediction with Hybrid ARIMA-SVMs Models

**DOI:** 10.1155/2014/567246

**Published:** 2014-02-27

**Authors:** Wei Ming, Yukun Bao, Zhongyi Hu, Tao Xiong

**Affiliations:** Department of Management Science and Information Systems, School of Management, Huazhong University of Science and Technology, Wuhan 430074, China

## Abstract

The hybrid ARIMA-SVMs prediction models have been established recently, which take advantage of the unique strength of ARIMA and SVMs models in linear and nonlinear modeling, respectively. Built upon this hybrid ARIMA-SVMs models alike, this study goes further to extend them into the case of multistep-ahead prediction for air passengers traffic with the two most commonly used multistep-ahead prediction strategies, that is, iterated strategy and direct strategy. Additionally, the effectiveness of data preprocessing approaches, such as deseasonalization and detrending, is investigated and proofed along with the two strategies. Real data sets including four selected airlines' monthly series were collected to justify the effectiveness of the proposed approach. Empirical results demonstrate that the direct strategy performs better than iterative one in long term prediction case while iterative one performs better in the case of short term prediction. Furthermore, both deseasonalization and detrending can significantly improve the prediction accuracy for both strategies, indicating the necessity of data preprocessing. As such, this study contributes as a full reference to the planners from air transportation industries on how to tackle multistep-ahead prediction tasks in the implementation of either prediction strategy.

## 1. Introduction

Forecasting is an important component of planning in any enterprise, but it is particularly critical in airline revenue management because of the direct influence forecasts have on the booking limits that determine airline profits. Unfortunately, the passengers behaviors and other complicating factors make air passengers traffic prediction extremely difficult.

In the past decades, academic researchers and practitioners have made many contributions to air passengers traffic prediction. Most of the prediction models abounded in the literature can mainly be classified into two categories, namely, econometric models (casual model) and time series models. In the econometrics community, traditional research has been focusing on the economic factors of the take-off and landing point, such as population, income, and price [1–5]. It should be noted that most econometric models aimed to reveal the underlying relationship between air passengers traffic flow and selected variables such as geoeconomic and service-related factors. Due to the complexity of econometric modeling in variables selection, time series approach is a promising alternative in air passengers traffic prediction [6]. However, these traditional time series methods always do not work in practice. The main reason is that the underlying assumption of these traditional time series methods is linearity and they cannot capture the nonlinear patterns hidden and recognize the irregularity well. Recent research focuses on modeling time series with complex nonlinearity, dynamic variation, and high irregularity [7–14].

Recently, the hybrid modeling approach integrating ARIMA, the most widely used linear model, and SVM, the well-established computational intelligence technique, has been proposed and justified in various areas [15–17]. Pai established a hybrid ARIMA and support vector machines model for stock price forecasting. Chen proposed a hybrid methodology that exploits the unique strength of the SARIMA (seasonal autoregressive integrated moving average) model and the support vector machines model in forecasting Taiwan's machinery industry production values. And Guo developed a hybrid Seasonal Autoregression Integrated Moving Average and Least Square Support Vector Machine (SARIMA-LSSVM) model to predict the mean monthly wind speed in Hexi Corridor, which shows that the developed method is simple and quite efficient. Additionally, Zhang combined the ARIMA and feedforward neural networks models in forecasting [18].

This hybrid approach arises from the fact that real-world time series are rarely pure linear or nonlinear but containing both linear and nonlinear patterns, and neither ARIMA nor SVM can be adequate in modeling and forecasting time series since the ARIMA model cannot deal with nonlinear relationships while the SVM model alone is not able to handle both linear and nonlinear patterns equally well. As such, this hybrid approach can take full advantages of the unique strength of ARIMA and SVM models in linear and nonlinear modeling, respectively. However, an important point to note from the past studies mentioned above is their preoccupation with one-step-ahead forecasting rather than the multistep-ahead one. In one-step-ahead case, the predictor uses all or some of the observations to estimate a variable of interests for the time step immediately following the latest observation. However, it provides no information as to the long-term future behavior of air passengers traffic.

In summary, this study extends the well-established ARIMA-SVMs model into the case of multistep-ahead prediction of air passengers traffic using the two modeling strategies, including iterated strategy and direct strategy and then goes a step forward by investigating the effectiveness of data preprocessing approaches, such as deseasonalization and detrending, along with the two strategies. The monthly air passengers traffic series from four airlines are used for the purpose of validation. The experimental results are judged on the basis of the prediction accuracy and the computational cost. The first contribution of this paper is that the proposed modeling framework is capable of capturing the complex dynamic of air passenger traffic considered, resulting in a more accuracy multistep-ahead prediction. The second contribution is to provide the first strong empirical evidence within the air passengers traffic literature on whether the superiority of ARIMA-SVMs modeling framework holds consistently in the case of multistep-ahead prediction. Last but not least, the third contribution is to provide the experimental evidence on the performance of different multistep-ahead prediction strategies, which may shed lights on the selection of modeling strategy while facing the different time horizons upon prediction.

The rest of this paper is organized as follows: Section 2 describes the methodologies used in this study including ARIMA and SVM along with the proposed hybrid methodology and multistep-ahead prediction strategies used in this study.  Section 3 details the research design on data source, data preprocessing, input selection, the selected counterparts for comparison, statistical criteria, methodological implementations, and experiment procedures. Following that, in Section 4, the experimental results are discussed.  Section 5 finally concludes this paper.

## 2. Methodologies

### 2.1. ARIMA-SVM Model

#### 2.1.1. ARIMA Model

ARIMA (*p*, *d*, *q*) is called Autoregressive Integrated Moving Average model. It is one of the basic models of Box-Jenkins modeling method. AR refers to autoregressive part of the model and *p* is the autoregressive order. MA is moving average and *q* is the term of moving average. *d* is the difference frequency before a time sequence become smooth.

Give a random sequence {*x*, *t* = 0,1,…}, *Bx*_*t*_ = *x*_*t*−1_, *B* is called as lagging operator. ∇ = 1 − *B*, ∇ is called as difference operator. If nonnegative integer *d* existed,

(1) $$\begin{array}{c} \hat{ο}\left(B\right)\nabla^{d}x_{t}=\theta \left(B\right)a_{t}, \end{array}$$

where $\hat{ο}(B)=1-{\hat{\text{o}}}_{1}B-{\hat{\text{o}}}_{2}B^{2}-\cdots -{\hat{\text{o}}}_{p}B^{p}$, *θ*(*B*) = 1 − *θ*_1_ *B* − *θ*_2_ *B* ^2^ − ⋯−*θ*_*q*_ *B* ^*q*^, |*B*| ≤ 1, and $\hat{ο}(B)$ and *θ*(*B*) are coprime, ${\hat{ο}}_{p}\theta_{p}\neq 0$, {*a*_*t*_} is white noise sequence, *Ea*_*t*_ = 0, *Ea*_1_^2^ = *σ* ^2^ < *∞*, then {*x*_*t*_} is called autoregressive and moving average sequence.

The basic idea of this model is to regard the time series as a random sequence and to use a certain mathematical model to describe the sequence approximately. Once the model is identified, we can use the past value to predict the future.

The basic steps are as follows.

1. Identify smooth sequence using scatter chart, autocorrelation function, partial autocorrelation function, and ADF unit root test.
2. Do smooth processing on nonstationary sequence such as difference.
3. Establish the model according to the time-series model identification rules. If the partial correlation function of a smooth sequence is censored and the autocorrelation function is trailing, sequence can be concluded for the AR model. If the partial correlation of a smooth sequence is trailing and the autocorrelation function is censored, it will be determined for MA model. And if both partial correlation function and autocorrelation function are trailing, ARMA model is for the sequence.
4. Estimate parameters and justify whether the statistical test is significant.
5. Do hypothesis test and diagnose whether the sequence is the white noise.
6. Predict with the model established.

#### 2.1.2. Support Vector Machine (SVM) Model

As a new method based on structural risk minimization principle, support vector machine is superior to those methods based on experience risk minimization principle. It can be converted to a salvation of a convex quadratic programming and make sure the extreme solution is the global optimal solutions. Support vector machines can handle higher dimension data better even with a relatively low amount of training samples and has a very good generalization. The models select limit support vectors from input data and so have a fast processing speed.

For linear and regressive data set {*x*_*i*_, *y*_*i*_}, the SVM regression function is formulated as follows:

(2) $$\begin{array}{c} f\left(x\right)=\omega^{T}x+b. \end{array}$$

The coefficients *ω* and *b* are estimated by minimizing

(3) $$\begin{array}{c} \frac{1}{2}\omega^{T}\omega +C\frac{1}{n}\overset{n}{\underset{i=1}{\sum }}L_{\varepsilon }\left(y_{i},f\left(x_{i}\right)\right), \end{array}$$

where *L*_*ε*_ is called the *ε*-intensive loss function and is formulated as follows:

(4) $$\begin{array}{c} L_{\varepsilon }\left(y,f\left(x\right)\right)=\{\begin{array}{cc} 0 & \text{if}\;\left|y-f\left(x\right)\leq \varepsilon \right|\\ \left|y-f\left(x\right)\right| & \text{others.} \end{array} \end{array}$$

By introducing slack variables *ξ* and ξ*, Equation (3) can be transformed to the following constrained formulation:

(5) $$\begin{array}{c} \min\frac{1}{2}\omega^{T}w+C\overset{n}{\underset{i=1}{\sum }}\left(\xi_{i}+\xi_{i}^{*}\right) \end{array}$$

subject to

(6) $$\begin{array}{c} \omega x_{i}+b_{i}-y_{i}\leq \varepsilon +\xi_{i}^{*}\\ -\omega x_{i}-b_{i}+y_{i}\leq \varepsilon +\xi_{i}\\ \xi_{i},\xi_{i}^{*}\geq 0\\ i=1,2,\ldots ,N. \end{array}$$

When solving the above formula, we always use dual theory to transform it into a convex quadratic programming problem. Introducing Lagrange equation, (5) changed into the following form:

(7) min⁡  12∑i,j=1n(αi∗−αi)(αj∗−αj)xiTxj−∑i=1nαi∗(yi−ε)−αi(yi+ε)

subject to

(8) $$\begin{array}{c} \overset{n}{\underset{i=1}{\sum }}\left(\alpha_{i}-\alpha_{i}^{*}\right)=0,\;\alpha_{i},\alpha_{i}^{*}\in \left[0,C\right]. \end{array}$$

When the data set cannot be regressed linearly, we also map them to a high dimension feature space and make linear regress. Then the formulation is as follows:

(9) min⁡⁡12∑i,j=1n(αi∗−αi)(αj∗−αj)Φ(xi)TΦ(xj)  −∑i=1nαi∗(yi−ε)−αi(yi+ε)

subject to

(10) $$\begin{array}{c} \overset{n}{\underset{i=1}{\sum }}\left(\alpha_{i}-\alpha_{i}^{*}\right)=0,\;\alpha_{i},\alpha_{i}^{*}\in \left[0,C\right]. \end{array}$$

Let *K*(*X*_*i*_, *X*_*j*_) = (Φ(*X*_*i*_) · Φ(*X*_*j*_)) = Φ^*T*^(*X*_*j*_)Φ(*X*_*i*_); *K*(*x*, *x*) is the inner product of feature space and is called kernel function. Any symmetric function which satisfies Mercer theory can be kernel function. There are some common kernel functions:

 polynomial kernel function: *K*(*x*_*i*_, *x*_*j*_) = (*x* ^*T*^ *x*_*i*_ + 1)^*p*^,
 RBF kernel function: *K*(*x*_*i*_, *x*_*j*_) = exp⁡(−*γ*||*x*_*i*_−*x*_*j*_||^2^),
 sigmoid kernel function: *K*(*x*_*i*_, *x*_*j*_) = th(*β*_0_ *x* ^*T*^ *x* + *β*_1_).

According to Karush-Kuhn-Tucker theory, the final SVM regression function can be the following form:

(11) $$\begin{array}{c} f\left(x\right)=\overset{n}{\underset{i=1}{\sum }}\left(\alpha_{i}-\alpha_{i}^{*}\right)K\left(x_{i},x\right)+b. \end{array}$$

#### 2.1.3. ARIMA-SVMs Model

Air passenger traffic demand is always affected by various factors such as economy, weather, and important events. Besides seasonality and long trend, there are always some irregular fluctuations which cannot be easily captured. ARIMA and SVM both have advantages in capturing linear or nonlinear patterns. So the hybrid methodology that has both linear and nonlinear modeling capabilities can be a good strategy for practical use. By combining different models, different aspects of the underlying patterns may be captured.

It is reasonable to consider a time series to be composed of a linear autocorrelation structure and a nonlinear component. That can then be represented as follows:

(12) $$\begin{array}{c} y_{t}=L_{t}+N_{t}, \end{array}$$

where *L*_*t*_ denotes the linear component and *N*_*t*_ denotes the nonlinear component. These two components have to be estimated from the data. First, we let ARIMA model the linear component, then the residuals from the linear model will contain only the nonlinear relationship. Let *e*_*t*_ denote the residual at time *t* from the linear model, and ${\hat{L}}_{t}$ denotes the forecast value of the ARIMA model, then

(13) $$\begin{array}{c} e_{t}=y_{t}-{\tilde{L}}_{t}. \end{array}$$

Zhang illustrated a linear model is not sufficient if there are still linear correlation structures left in the residuals. Moreover, even if a model has passed diagnostic checking, the model may still not be adequate in that nonlinear relationships have not been appropriately modeled [18]. So by modeling residuals using SVM, nonlinear relationships can be discovered. That can be represented as follows:

(14) $$\begin{array}{c} e_{t}=f\left(e_{t-1},e_{t-2},\ldots ,e_{t-n}\right)+\varepsilon_{t}, \end{array}$$

where *f* is a nonlinear function determined by SVM and *ε*_*t*_ is the random error. Therefore, the combined forecast is

(15) $$\begin{array}{c} y_{t}={\tilde{L}}_{t}+{\tilde{N}}_{t}, \end{array}$$

where ${\tilde{N}}_{t}$ is the forecast value of the SVM model. In summary, the ultimate forecast value consists of linear forecast part and nonlinear forecast part.

### 2.2. Iterated Strategy and Direct Strategy

Multistep-ahead time series forecasting can be described as an estimation on future time series *φ*_*N*+*h*_, (*h* = 1,2,…, *H*), while *H* is an integer and more than one, given the current and previous observation *φ*_*t*_, (*t* = 1,2,…, *N*). In the present study, iterated strategy and direct strategy are frequently selected for multistep-ahead forecasting. For each selected strategies, there are a large number of variations proposed in the literatures, and it would be a hopeless task to consider all existing varieties. Our guideline was therefore to consider the basic version of each strategy (without the additions, or the modifications proposed by some other researchers). The reason for selecting the following two strategies is that they are some of the most commonly used strategies. The following subsection presents a detailed definition of the selected strategies.

#### 2.2.1. Iterated Strategy

The first is named as the iterated strategy by Chevillon [19] and is often advocated in standard time series textbooks [20, 21]. This strategy constructs a prediction model by means of minimizing the squares of the in-sample one-step-ahead residuals and then uses the predicted value as a “known” input to forecast the next point and continues in this manner until reaching the horizon.

In more detail, iterated strategy first embeds the original series into an input-output format:

(16) $$\begin{array}{c} D={\left\{\left(x_{t},y_{t}\right)\in \left(R^{m}\times R\right)\right\}}_{t=d}^{N}, \end{array}$$

where *x*_*t*_ ⊂ {*φ*_*t*_,…, *φ*_*t*−*d*+1_}, *y*_*t*_ = *φ*_*t*+1_.

Then the iterated prediction strategy learns one-step-ahead prediction model:

(17) $$\begin{array}{c} \varphi_{t+1}=f\left(x_{t}\right)+\omega , \end{array}$$

where *ω* denotes the additive noise.

After the learning process, the estimation of the *H* next values is returned by

(18) φ^t+h ={f^(φt,φt−1,…,φt−d+1)if  h=1f^(φ^t+h−1,φ^t+1,φt,…,φt−d+h)if  h∈[2,…,d]f^(φ^t+h−1,…,φ^t+h−d)if  h∈[d+1,…,H].

#### 2.2.2. Direct Strategy

In contrast to the iterated strategy which uses a single model, the other commonly applied strategy, namely, direct strategy first suggested by Cox [22], constructs a set of prediction models for each horizon using only its past observations, where the associated squared multistep-ahead errors are minimized. Direct strategy estimates *H* different models between the inputs and the outputs to predict {*φ*_*N*+*h*_, *h* = 1,2,…, *H*}, respectively, which requires a sharply increased computational expense, especially in the case of a longer prediction horizon [23]. The direct strategy first embeds the original series into *H* datasets

(19) D1={(xt,yt1)∈(Rm×R)}t=dN,  ⋮DH={(xt,ytH)∈(Rm×R)}t=dN,

where *x*_*t*_ ⊂ {*φ*_*t*_,…, *φ*_*t*−*d*+1_}, *y*_*th*_ = *φ*_*t*+*h*_.

Then, the direct prediction strategy learns *H* direct models on *D*_*h*_ = {*D*_1_,…, *D*_*H*_}, respectively. Consider

(20) $$\begin{array}{c} \varphi_{t+h}=f_{h}\left(x_{t}\right)+\omega_{h},\;h\in \left\{1,\ldots ,H\right\}, \end{array}$$

where *ω* denotes the additive noise.

After the learning process, the estimation of the *H* next values is returned by

(21) $$\begin{array}{c} \varphi_{t+h}={\hat{f}}_{h}\left(\varphi_{t},\varphi_{t-1},\ldots ,\varphi_{t-d+1}\right),\;h\in \left\{1,\ldots ,H\right\}. \end{array}$$

### 2.3. ARIMA-SVMs Model for Multistep-Ahead Prediction

In this study, we consider *H*-step ahead forecasting by ARIMA-SVMs model. The proposed methodology of the multistep-ahead prediction hybrid system consists of two stages. In the first stage, an ARIMA model is used to analyze the linear part of the problem for each step. In the second stage, SVMs model is developed to model the residuals from the ARIMA model. Since the ARIMA model cannot capture the nonlinear structure of the data, the residuals of linear model will contain information about the nonlinearity. Assuming *y*_*t*_ (*t* = 1,2,…, *n*) is the original data set and according to ARIMA-SVMs model, *y*_*t*_ includes the linear part *L*_*t*_ and nonlinear part *N*_*t*_, namely, *y*_*t*_ = *L*_*t*_ + *N*_*t*_. First of all, *H*-step ahead prediction by ARIMA model will be employed and obtain the *H*-step ahead forecasted value ${\overset{\sim }{L}}_{t+h}$, then for each time *t*, the residuals of *y*_*t*+*h*_ can be obtained by $\varepsilon_{t+h}=y_{t+h}-{\overset{\sim }{L}}_{t+h}$. And for nonlinear time series *ε*_*t*_ (*t* = 1,2,…, *n*), *H*-step ahead prediction by SVMs model will be employed and obtain the *H*-step ahead forecasted values ${\overset{\sim }{N}}_{t+h}$, the ultimate forecasted time series for *y*_*t*+*h*_ is ${\overset{\sim }{y}}_{t+h}$, which consist of ${\overset{\sim }{L}}_{t+h}$ and ${\overset{\sim }{N}}_{t+h}$. In this research, two multistep-ahead forecasting strategies will be employed, namely, iterated strategy and direct strategy, and it should be noted that if the multistep-ahead strategy has been selected for linear part, the same strategy should be employed in nonlinear part; therefore, the model should be ARIMA (iterated strategy)-SVMs(iterated strategy) or ARIMA(direct strategy)-SVMs(direct strategy).

**Figure 1:**
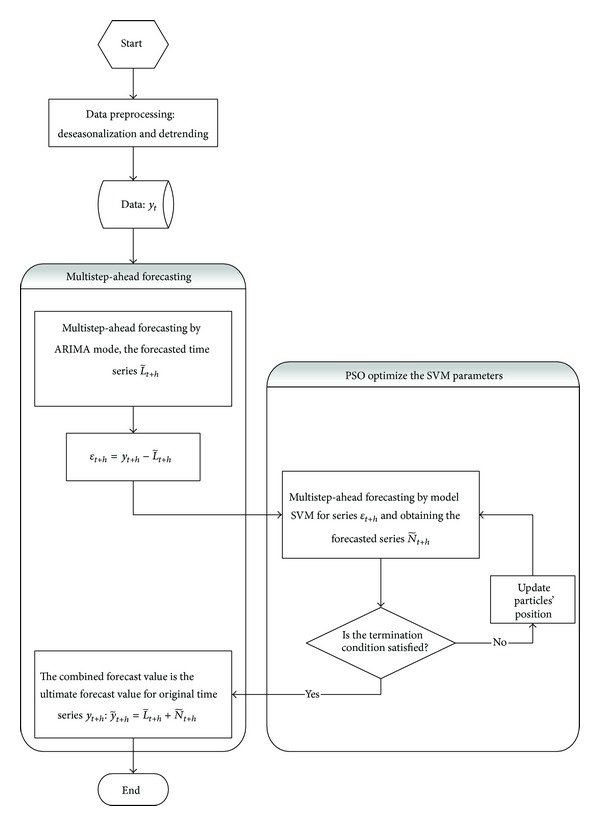
The flowchart of proposed multistep-ahead PSO-ARIMA-SVMs modeling framework.

The flowchart of proposed multistep-ahead ARIMA-SVMs modeling framework is depicted in Figure 1. As shown in Figure 1, the proposed modeling framework includes three parts: data preprocessing, multistep-ahead prediction, and PSO module for SVMs parameters optimization (details on this parameters tuning module can be found in Section 3.3). In data preprocessing procedure, linear transference, deseasonalization, and detrending are performed on the data, and then the multistep-ahead forecasting model based on ARIMA-SVMs is adopted; in addition, in nonlinear forecasting part, the parameter optimization algorithm PSO is employed to optimize the parameters of SVM.

## 3. Research Design

### 3.1. Data and Preprocessing

#### 3.1.1. Data

In this study, four monthly air passenger traffic series of American airlines are chosen as experimental samples, namely, American Airlines, Delta Air Lines, Southwest Airlines, and United Airlines. The data are freely obtained from the Bureau of Transportation Statistics, U.S. Department of Transportation (http://www.bts.gov/). The main reason of selecting these four airlines is that these airlines are famous in America and they represent the development trend of air industry in America.

**Figure 2:**
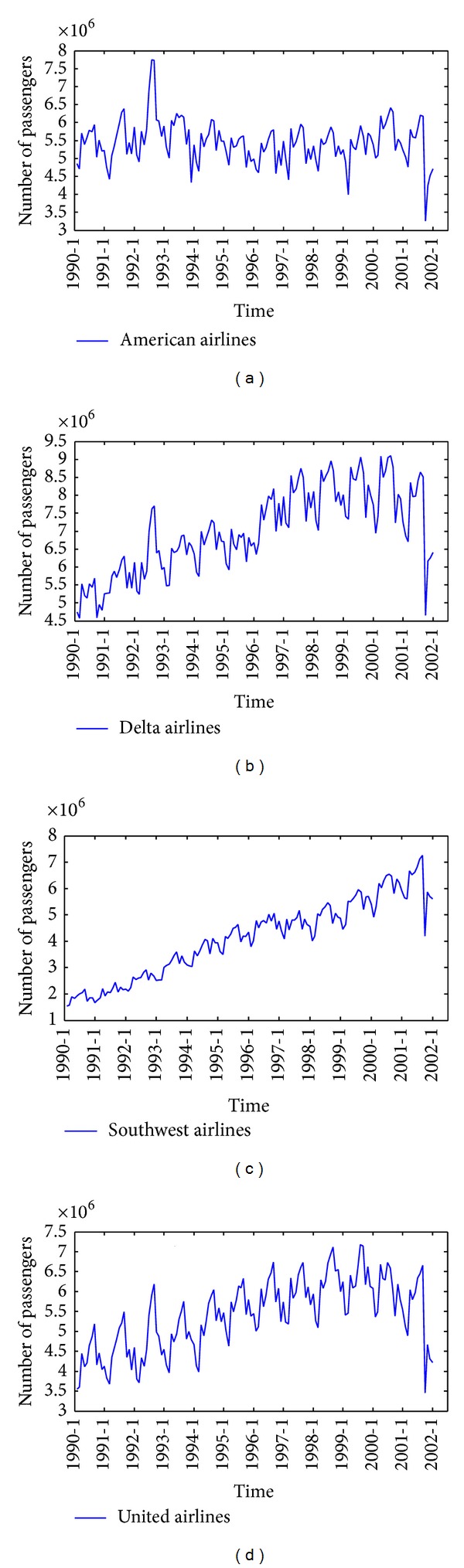
(a) American Airlines data. (b) Delta Air Lines data. (c) Southwest Airlines data. (d) United Airlines data.

Considering the Severe Acute Respiratory Syndrome (SARS) which happened in 2003 and brought a heavy blow to American air industry, the four sampling data series cover a period from January 1990 to December 2001, each with a total of 144 observations. The original data of these four airlines are shown in Figures 2(a) to 2(d).

The descriptive statistics details of all the four data series are summarized in Table 1.

#### 3.1.2. Preprocessing

Normalization is a standard requirement for time series modeling and prediction with neural network. Thus, the data sets are first scaled by linear transference to map onto a range of [0, 1]. As mentioned in Section 1, the air passengers traffic data are monthly and with strong seasonal components and trend patterns. Therefore, after the linear transference, deseasonalization and detrending are performed on the data. Deseasonalization is performed by means of the revised multiplicative seasonal decomposition presented in [24]. In addition, detrending is performed by fitting a polynomial time trend and then subtracting the estimated trend from the series when trends are detected by the Mann-Kendall test [25].

### 3.2. Input Selection

Filter method was employed for input selection in this study. In the case of the filter method, the best subset of inputs is selected *a priori *based only on the dataset. The input subset is chosen by a predefined criterion, which measures the relationship of each subset of input variables with the output [26]. Specifically, in terms of input selection criteria, the partial mutual information [27] is used for the prediction models. Mutual information (MI) is a commonly adopted measure of dependence between variables and has been widely used for input selection [26]. However, this raises a major redundancy issue because the MI criterion does not account for the interdependency between candidate variables. To address this issue, Sharma [27] developed an improved algorithm that exploits the concept of partial mutual information (PMI), which is the nonlinear statistical analog of partial correlation. The definitions of PMI are shown as follows:

(22) $$\begin{array}{c} \text{PMI}=\iint f_{X^{\prime },Y^{\prime }}\left(x^{\prime },y^{\prime }\right)\log_{e}\left[\frac{f_{X^{\prime },Y^{\prime }}\left(x^{\prime },y^{\prime }\right)}{f_{X^{\prime }}\left(x^{\prime }\right)f_{Y^{\prime }}\left(y^{\prime }\right)}\right]dx^{\prime }dy^{\prime }, \end{array}$$

where

(23) $$\begin{array}{c} x^{\prime }=x-E\left[x\;|\;z\right],\\ y^{\prime }=y-E\left[y\;|\;z\right], \end{array}$$

where *X*′ and *Y*′ are generalized to represent time series *x*(*t*) and lagged time *x*(*t* − *i*) with time step *i*  (*i* ≤ *d*) conditional on *Z* which is a set of remaining time-lag variables. In performing the PMI, the input variable with highest conditional PMI value at every iteration can be added to the inputs set.

### 3.3. Parameters Selection with PSO

In this study, RBF has been chosen as the kernel function for SVM models, and thus the parameters *ε*, *γ*, and *C* are to be optimized based on the training sets. Despite the new variants of PSO for parameter tuning such as in [28], in this study, we chose the standard PSO algorithm to tune the parameters with the lowest MAPE on training sets.

PSO based operators are used to explore the search space. Particle Swarm Optimization (PSO) is a population-based metaheuristic that simulates social behavior such as birds flocking to a promising position to achieve precise objectives (e.g., finding food) in a multidimensional space by interacting among them [29]. To search for the optimal solution, each particle adjusts their flight trajectories by using the following updating equations:

(24) $$\begin{array}{c} v_{id}^{t+1}=w\times v_{id}^{t}+c_{1}\times r_{1}\times \left(p_{id}-x_{id}^{t}\right)+c_{2}\times r_{2}\times \left(p_{gd}-x_{id}^{t}\right),\\ x_{id}^{t+1}=x_{id}^{t}+v_{id}^{t+1}, \end{array}$$

where *c*_1_, *c*_2_ ∈ *ℜ* are acceleration coefficients, *w* is inertia weight, and *r*_1_ and *r*_2_ are random numbers in the range of [0,1]. *v*_*id*_^*t*^ and *x*_*id*_^*t*^ denote the velocity and position of the *i*th particle in *d*th dimension at *t*th iteration, respectively. *p*_*id*_ is the value in dimension *d* of the best parameters combination (a particle) found so far by particle *i*. *p*_*i*_ = 〈*p*_*i*1_,…, *p*_*iD*_〉 is called personal best (*pbest*) position. *p*_*gd*_ is the value in dimension *d* of the best parameters combination (a particle) found so far in the swarm (*P*); *p*_*g*_ = 〈*p*_*g*1_,…, *p*_*gD*_〉 is considered as the global best (*gbest*) position. Note that each particle takes individual (*lbest*) and social (*gbest*) information into account for updating its velocity and position.

In the search space, particles track the individual's best values and the best global values. The process is terminated if the number of iterations reaches the predetermined maximum number of iterations.

### 3.4. The Selected Counterparts for Comparison

Single SVR and monthly ARIMA are selected as counterparts for the purpose of comparison. It should be noted the reason for selecting single SVR and monthly ARIMA is to justify the effectiveness of ARIMA-SVM modeling framework. Additionally, the performances on both one-step-ahead (prediction horizon *H* = 1) and multistep-ahead (prediction horizon *H* = 2,4, 6,8, 12,18,24) prediction are compared across all the models to provide more evidences for justification. Note that the iterated strategy and direct strategy are employed in this study for comparison.

### 3.5. Statistical Criteria and Rank-Based Measures

To compare the effectiveness of the different prediction models, no single accuracy measure can capture all the distributional features of the errors when summarized across data series. For each forecast horizon *h*, here, we consider three forecast accuracy measures. The first is the mean absolute percentage error (MAPE) defined as (25), and the second is symmetric mean absolute percentage error (SMAPE) defined as (26), and the last accuracy measure is the mean absolute scaled error (MASE), defined as (27). MAPE has the advantage of being scale-independent and so is frequently used to compare forecast performance across different datasets. Moreover, the MAPE also has the disadvantage that it puts a heavier penalty on positive errors than on negative errors. MASE has recently been suggested by Hyndman and Koehler [30] as a means of overcoming observation and errors around zero existing in some measures. The MASE has some features which are better than the SMAPE, which has been criticized for the fact that its treatment of positive and negative errors is not symmetric [31]. However, the MAPE and SMAPE are still used in this study due to their popularity in forecasting literature. The smaller the values of MAPE, SMAPE, and MASE, the closer the predicted time series values to the actual values. Consider

(25) $$\begin{array}{c} \text{MAP}{\text{E}}_{h}=\frac{1}{M\cdot T}\overset{M}{\underset{m=1}{\sum }}\;\overset{T}{\underset{t=1}{\sum }}\left(100\left|\frac{x_{m}^{h}\left(t\right)-{\hat{x}}_{m}^{h}\left(t\right)}{x_{m}^{h}\left(t\right)}\right|\right), \end{array}$$

(26) $$\begin{array}{c} {\text{SMAPE}}_{h}=\frac{1}{M\cdot T}\overset{M}{\underset{m=1}{\sum }}\;\overset{T}{\underset{t=1}{\sum }}\left(100\left|\frac{x_{m}^{h}\left(t\right)-{\hat{x}}_{m}^{h}\left(t\right)}{\left(\left|x_{m}^{h}\left(t\right)\right|+\left|{\hat{x}}_{m}^{h}\left(t\right)\right|\right)/2}\right|\right), \end{array}$$

(27) MASEh =1M·T∑m=1M ∑t=1T|xmh(t)−x^mh(t)(1/(N−1))∑i=2N|xm(i)−xm(i−1)||,

where *x*_*m*_(*t*) denotes the observation at period *t* for time series *m*, ${\hat{x}}_{m}(t)$ denotes the forecasted value of *x*_*m*_(*t*), *M* is the number of time series (in this case, *M* = 4), *T* is the number of observation in the hold-out sample (in this case, *T* = 48), and *N* is the number of observation in the estimation sample for time series *m*.

In this study, we repeat running each model fifty times for airline passengers traffic dataset to even out the fluctuations. Then each of the fifty runs will produce a SMAPE for all 4 time series. Next, the mean and standard deviation of these fifty SMAPE are calculated and listed in the tables for examining the performance of different models. Similarly, the mean and standard deviation of MASE are also computed. Note that the error measures are computed after rolling back of the preprocessing step performed, such as deseasonalization and detrending.

Following the experimental settings in [32], we also conduct a number of statistical tests to compare each model based on the obtained fifty SMAPE and MASE at the 0.05 significance level. For each prediction horizon (*H* = 1 and 24) and performance measure (i.e., MAPE, SMAPE, and MASE), we perform a one-way analysis of variance (ANOVA) procedure to determine if there exists statistically significant difference among the eight models in out-of-sample forecasting. Then, to further identify the significant difference between any two models, the Tukey honestly significant difference (HSD) test [33] is employed to compare all pairwise difference simultaneously. Note that Tukey HSD test is a post-hoc test; this means that a researcher should not perform Tukey HSD test unless the results of ANOVA are positive. A rank-based performance measure termed as multiple comparisons with the best (MCB) [34] is used to test whether some models perform significantly worse than the best model.

### 3.6. Implementations

LibSVM (version 2.86) [35] is employed for SVR modeling here. We select the Radial basis function (RBF) as the kernel function in the prediction models. To determine the hyperparameters, namely, *C*, *ε*, *γ* (in the case of RBF as the kernel function), the PSO algorithm is employed in the current study. Due to its simplicity and generality as no important modification was made for applying it to model selection, PSO has been recently established for parameter determination of SVR. In solving hyperparameter selection by the PSO, each particle is requested to represent a potential solution (*C*, *ε*, *γ*). Fortunately, several empirical and theoretical studies have been performed about the parameters of PSO from which valuable information can be obtained [36]. In this study, the parameters are determined according to the recommendations in these studies and selected based on the prediction performance and computational time in a trial-error fashion. Through experiments, the parameter values of PSO are set as follows. Both the cognitive and interaction coefficients are set to 2. The swarm size and number of iterations are set to be 10 and 50, respectively.

For monthly ARIMA estimation, the automatic model selection algorithm proposed by Hyndman and Khandakar and implemented in the R software package “forecast”^5^ is used in this study. Based on these, we develop our proposed hybrid ARIMA-SVMs programs in Matlab, which is available upon request.

### 3.7. Experimental Procedure

**Figure 3:**
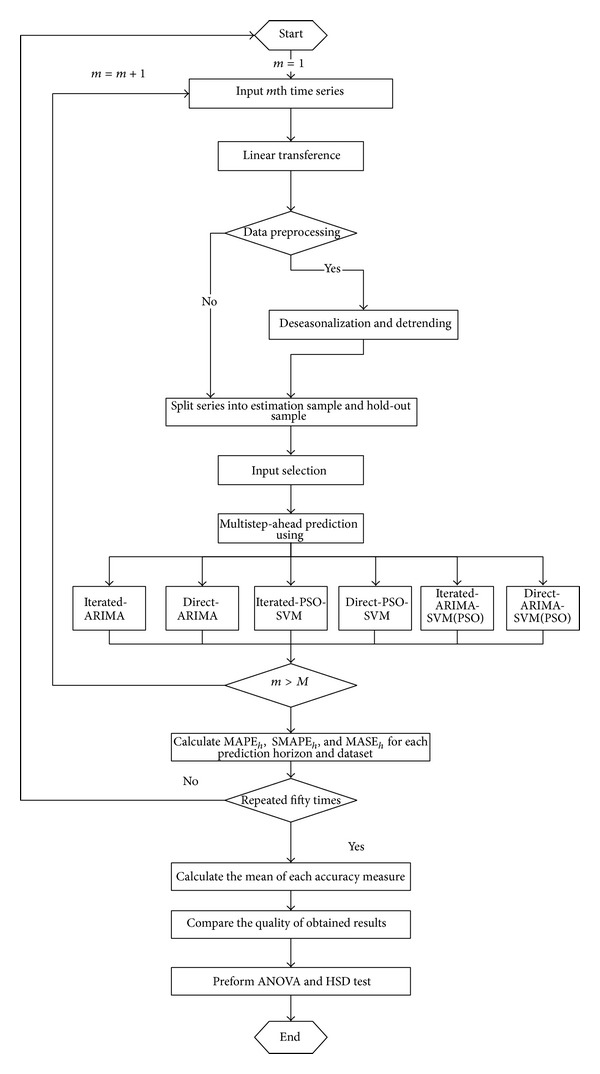
Experiment procedure.

Figure 3 shows the experimental procedure using the real time series. Each series is split into the estimation sample and the hold-out sample firstly. Then, the input selection and model selection for each series are conducted using aforementioned filter method, PSO algorithm, and fivefold cross-validation with iterated and direct strategies. Finally, the attained models are tested for hold-out samples, the measures MAPE_*h*_, SMAPE_*h*_, and MASE_*h*_ are computed for each prediction horizon *h* (in our case *h* = 1,2,…, 24) over four time series. Furthermore, the modeling process for each series is repeated fifty times. Upon the termination of this loop, performance of the examined models with selected strategies at each prediction horizon is judged in terms of the mean, average by fifty, of the MAPE_*h*_, SMAPE_*h*_, and MASE_*h*_. Analysis of variance (ANOVA) test procedures are used to determine if the means of performance measures are statistically different among the five models for each prediction horizon and dataset. If so, Tukey's honesty significant difference (HSD) tests [37] are then employed further to identify the significantly different prediction models in multiple pairwise comparisons at 0.05 significance level.

## 4. Results and Discussion

The forecasting performance of the hybrid ARIMA-SVMs and benchmarking method on the four airlines' air passenger series covered a period from January 1990 to December 2001 are shown in Table 2.

For each accuracy measure and prediction horizon, the strategies are rank order from 1 (the best) to 6 (the worst) in Table 2.

- As far as the comparison between ARIMA-SVMs with others benchmark methods is concerned, the results are mixing among the prediction measures examined. In terms of MAPE and the data without deseasonalization-detrending, I-ARIMA-SVM gets the best performance when horizon = 1, and D-SVM gets the best performance when horizon = 4, 8, 12 and in the average accuracy measures over the prediction horizon 1 to 24, while D-ARIMA-SVM gets the performance when horizon = 2, 6, 18, 24.
- It is obvious that applying deseasonalization and detrending approach for multistep-ahead prediction obtains better performances.

Following the experimental procedure presented in Figure 3, an ANOVA procedure is performed to determine if there exists a statistically significant difference among the six modeling strategies in the hold-out sample for each of the performance measures and the prediction horizon. Furthermore, to identify the significant difference between any two strategies, the Tukey's HSD test is used to compare all pairwise differences simultaneously. Tukey's HSD test is a post-hoc test, meaning that Tukey's HSD test should not be performed unless the results of the ANOVA procedure are positive.  Table 3 shows the results of the multiple comparison tests for three datasets. Several observations can be made from Table 3.

- As for the comparison between ARIMA-SVMs and the benchmark methods, the difference in prediction performance is significant at the 0.05 level and ARIMA-SVMs is better than SVM and ARIMA.
- Concerning the current two multistep-ahead forecasting strategies, the direct strategy significantly outperforms the iterated strategy for the majority of prediction horizons.

During the experiments, we also found that when we try to improve the forecast accuracy of SVM, there will be an overfitting on training sets. That means, though we can get a better forecast performance of the SVM model, we cannot get a better forecast performance or even get a worse performance of the hybrid ARIMA-SVM model. Therefore, the optimal parameters of the hybrid model should be further researched.

## 5. Conclusion

Due to the complex and dynamic pattern with uncertainty, time series prediction still remains as one of the most challenging tasks in field of time series analysis. The hybrid ARIMA-SVMs prediction models have been established recently, which has present good performance in time series prediction. In this study, the hybrid ARIMA-SVMs has been employed in multistep-ahead prediction which is more complex and difficult than one-step-ahead. The contribution of this study is employing ARIMA-SVMs in multistep-ahead prediction; moreover, air passengers traffic data is used for this study and a large scale comparative study has been conducted for validation. Quantitative and comprehensive assessments are performed with the air passengers traffic data on the basis of the several prediction accuracies. Experimental results and comparisons demonstrate the superiority of the proposed ARIMA-SVMs modeling strategy for multistep-ahead time series prediction.

## Figures and Tables

**Table 1:** Descriptive statistics of all data series.

	Period	*N*	Min	Max	Mean	Std	Skewness		Kurtosis	
							Statistic	Std. error	Statistic	Std. error
American	90.01–01.12	144	3251642	7747239	5443463	597069	0.309	0.202	2.988	0.401
Delta	90.01–01.12	144	4565958	9104110	7003819	1168848	−0.073	0.202	−0.904	0.401
Southwest	90.01–01.12	144	1538109	7257119	4110732	1461296	−0.016	0.202	−0.969	0.401
UnitedAir	90.01–01.12	144	3449108	7176446	5416990	887173	−0.225	0.202	−0.791	0.401

**Table 2:** Prediction accuracy measures for hold-out sample.

Data preprocess	Strategy	Prediction horizon (*h*)						Average 1 − *h*			Average rank
		1	2	4	6	8	12	18	24	1–24	
Non-deseasonalization-detrending		MAPE									
	I-ARIMA	22.235	24.580	24.127	25.328	22.485	26.369	28.059	31.254	25.555	5.444
	I-SVM	7.079	10.549	13.013	13.580	11.768	10.045	18.510	10.506	11.881	3.889
	D-ARIMA	26.025	25.895	23.745	24.765	25.014	25.956	27.654	32.547	26.450	5.556
	D-SVM	7.096	**7.521 **	7.754*	8.353	7.140*	7.191*	9.553	9.830	8.055*	**2.111**
	I-ARIMA-SVM	6.291*	7.895	8.025	**8.281 **	8.781	8.943	**9.295 **	**9.728 **	8.405	2.444
	D-ARIMA-SVM	**6.341 **	7.213*	**7.984 **	8.061*	**8.632 **	**8.259 **	8.894*	9.621*	**8.126 **	1.556*
		SMAPE									
	I-ARIMA	22.204	24.319	23.547	24.412	21.541	25.849	26.462	30.146	24.810	5.444
	I-SVM	7.075	10.287	12.384	12.509	11.284	9.623	16.158	10.009	11.166	3.889
	D-ARIMA	25.884	24.998	23.017	24.251	24.932	25.687	26.199	31.968	25.867	5.556
	D-SVM	7.089	**7.452 **	7.580*	8.084	6.895*	6.997*	8.853	9.166*	7.765*	**1.889 **
	I-ARIMA-SVM	6.457*	7.644	7.995	7.543*	7.687	8.787	**8.783 **	9.964	8.108	2.444
	D-ARIMA-SVM	**6.549 **	6.956*	**7.885 **	**7.983 **	**7.564 **	**7.784 **	8.595*	**9.589 **	**7.863 **	1.778*
		MASE									
	I-ARIMA	10.024	11.581	10.992	12.067	11.125	12.694	14.553	16.005	12.380	5.333
	I-SVM	2.718	4.259	4.079	4.193	3.197	2.267*	3.847	3.282*	3.480	3.111
	D-ARIMA	12.099	11.867	11.261	11.794	11.803	12.043	13.216	17.258	12.668	5.667
	D-SVM	2.731	2.728	**2.607 **	2.605*	1.906*	2.759	**2.902 **	**4.964 **	**2.900 **	**2.222 **
	I-ARIMA-SVM	1.982*	1.968*	3.058	**2.953 **	2.051	3.352	3.872	5.942	3.147	2.778
	D-ARIMA-SVM	**2.058 **	**2.181 **	2.393*	3.186	**2.011 **	**2.421 **	2.643*	5.027	2.740*	1.889*

deseasonalization-detrending		MAPE									
	I-ARIMA	20.278	22.569	22.127	23.315	20.483	24.434	26.146	29.348	23.587	5.444
	I-SVM	5.150	8.566	11.041	11.595	9.793	8.138	16.625	8.628	9.942	3.889
	D-ARIMA	24.068	23.884	21.745	22.752	23.012	24.021	25.741	30.641	24.483	5.556
	D-SVM	5.276	**5.647 **	5.891*	6.477	5.275*	5.393*	7.777	8.061	6.225*	**2.111 **
	I-ARIMA-SVM	4.471*	6.021	6.162	**6.405 **	6.916	7.145	**7.519 **	**7.959 **	6.575	2.444
	D-ARIMA-SVM	**4.521 **	5.339*	**6.121 **	6.185*	**6.767 **	**6.461 **	7.118*	7.852*	**6.295 **	1.556*
		SMAPE									
	I-ARIMA	20.136	22.197	21.436	22.289	19.428	23.803	24.438	28.129	22.732	5.444
	I-SVM	5.242	8.400	10.509	10.621	9.406	7.813	14.370	8.228	9.324	4.000
	D-ARIMA	23.836	22.896	20.926	22.147	22.839	23.661	24.195	29.971	23.809	5.556
	D-SVM	5.233	**5.542 **	5.681*	6.173	4.994*	5.163*	7.042	7.362*	5.899*	1.778*
	I-ARIMA-SVM	4.601*	5.734	6.096	5.632*	5.786	6.953	6.972*	8.160	6.242	2.444
	D-ARIMA-SVM	**4.693 **	5.046*	**5.986 **	**6.072 **	**5.663 **	**5.950 **	**6.784 **	**7.785 **	**5.997 **	1.778*
		MASE									
	I-ARIMA	8.533	10.037	9.459	10.521	9.589	11.226	13.107	14.566	10.880	5.333
	I-SVM	1.747	3.235	3.066	3.167	2.181	1.319*	2.921	2.363*	2.500	3.333
	D-ARIMA	11.056	10.370	9.775	10.295	10.314	10.622	11.817	15.866	11.264	5.667
	D-SVM	1.657	1.600	1.490	1.475*	0.787*	1.707	**1.872 **	**3.941 **	**1.816 **	**2.111 **
	I-ARIMA-SVM	0.908*	0.840*	**1.941 **	**1.823 **	0.932	2.300	2.842	4.919	2.063	2.667
	D-ARIMA-SVM	**0.984 **	**1.053 **	1.276*	2.056	**0.892 **	**1.369 **	1.613*	4.004	1.656*	1.889*

Note: I-ARIMA means ARIMA model which employs iterated strategy; D-ARIMA means ARIMA model which employs direct strategy, the same as I-SVM, D-SVM, I-ARIMA-SVM, and D-ARIMA-SVM. For each column of table, the entry with the smallest value is set in boldface and marked with an asterisk, and the entry with second smallest value is set in boldface type.

**Table 3:** Multiple comparison results with ranked strategies for hold-out sample.

Data preprocess	Measure	Prediction horizon (*h*)	Rank of strategies										
			1		2		3		4		5		6
Non-deseasonalization-detrending	MAPE_*h*_	1	I-ARIMA-SVM	<	D-ARIMA-SVM	<*	I-SVM	<	D-SVM	<*	I-ARIMA	<	D-ARIMA
		2	D-ARIMA-SVM	<	D-SVM	<	I-ARIMA-SVM	<	I-SVM	<*	D-ARIMA	<	I-ARIMA
		4, 12	D-SVM	<	D-ARIMA-SVM	<*	I-ARIMA-SVM	<	I-SVM	<*	D-ARIMA	<	I-ARIMA
		6, 18	D-ARIMA-SVM	<*	I-ARIMA-SVM	<	D-SVM	<	I-SVM	<*	D-ARIMA	<	I-ARIMA
		8, 1–24	D-SVM	<	D-ARIMA-SVM	<	I-ARIMA-SVM	<	I-SVM	<*	I-ARIMA	<	D-ARIMA
		24	D-ARIMA-SVM	<*	I-ARIMA-SVM	<	D-SVM	<	I-SVM	<*	I-ARIMA	<	D-ARIMA
	SMAPE_*h*_	1	I-ARIMA-SVM	<	D-ARIMA-SVM	<*	I-SVM	<	D-SVM	<*	I-ARIMA	<	D-ARIMA
		2	D-ARIMA-SVM	<	D-SVM	<	I-ARIMA-SVM	<	I-SVM	<*	I-ARIMA	<	D-ARIMA
		4, 12	D-SVM	<	D-ARIMA-SVM	<	I-ARIMA-SVM	<*	I-SVM	<*	D-ARIMA	<	I-ARIMA
		6	I-ARIMA-SVM	<	D-ARIMA-SVM	<	D-SVM	<	I-SVM	<*	D-ARIMA	<	I-ARIMA
		8, 24, 1–24	D-SVM	<	D-ARIMA-SVM	<*	I-ARIMA-SVM	<*	I-SVM	<*	I-ARIMA	<	D-ARIMA
		18	D-ARIMA-SVM	<*	I-ARIMA-SVM	<	D-SVM	<	I-SVM	<	D-ARIMA	<*	I-ARIMA
	MASE_*h*_	1	I-ARIMA-SVM	<	D-ARIMA-SVM	<*	I-SVM	<	D-SVM	<*	I-ARIMA	<	D-ARIMA
		2	I-ARIMA-SVM	<	D-ARIMA-SVM	<*	D-SVM	<	I-SVM	<*	I-ARIMA	<	D-ARIMA
		4, 1–24	D-ARIMA-SVM	<	D-SVM	<	I-ARIMA-SVM	<*	I-SVM	<	I-ARIMA	<	D-ARIMA
		6	D-SVM	<	I-ARIMA-SVM	<	D-ARIMA-SVM	<*	I-SVM	<*	D-ARIMA	<	I-ARIMA
		8	D-SVM	<	D-ARIMA-SVM	<*	I-ARIMA-SVM	<	I-SVM	<*	I-ARIMA	<	D-ARIMA
		12	I-SVM	<	D-ARIMA-SVM	<	D-SVM	<	I-ARIMA-SVM	<	D-ARIMA	<	I-ARIMA
		18	D-ARIMA-SVM	<	D-SVM	<	I-SVM	<	I-ARIMA-SVM	<*	D-ARIMA	<	I-ARIMA
		24	I-SVM	<	D-SVM	<	D-ARIMA-SVM	<	I-ARIMA-SVM	<*	I-ARIMA	<	D-ARIMA

Deseasonalization-detrending	MAPE_*h*_	1	I-ARIMA-SVM	<	D-ARIMA-SVM	<*	I-SVM	<	D-SVM	<*	I-ARIMA	<	D-ARIMA
		2	D-ARIMA-SVM	<	I-ARIMA-SVM	<	D-SVM	<	I-SVM	<*	I-ARIMA	<	D-ARIMA
		4	D-SVM	<	D-ARIMA-SVM	<*	I-ARIMA-SVM	<*	I-SVM	<*	D-ARIMA	<	I-ARIMA
		6, 18, 24	D-ARIMA-SVM	<	I-ARIMA-SVM	<	D-SVM	<	I-SVM	<*	D-ARIMA	<	I-ARIMA
		8, 1–24	D-SVM	<	D-ARIMA-SVM	<*	I-ARIMA-SVM	<	I-SVM	<*	I-ARIMA	<*	D-ARIMA
		12	D-SVM	<	D-ARIMA-SVM	<*	I-ARIMA-SVM	<	I-SVM	<*	D-ARIMA	<	I-ARIMA
	SMAPE_*h*_	1	I-ARIMA-SVM	<	D-ARIMA-SVM	<	D-SVM	<	I-SVM	<*	I-ARIMA	<	D-ARIMA
		2	D-ARIMA-SVM	<	D-SVM	<	I-ARIMA-SVM	<	I-SVM	<*	I-ARIMA	<	D-ARIMA
		4, 12	D-SVM	<	D-ARIMA-SVM	<*	I-ARIMA-SVM	<*	I-SVM	<*	D-ARIMA	<	I-ARIMA
		6, 18	I-ARIMA-SVM	<	D-ARIMA-SVM	<*	I-SVM	<	D-SVM	<*	I-ARIMA	<	D-ARIMA
		8, 24, 1–24	D-SVM	<	D-ARIMA-SVM	<	I-ARIMA-SVM	<*	I-SVM	<*	I-ARIMA	<	D-ARIMA
	MASE_*h*_	1, 2	I-ARIMA-SVM	<	D-ARIMA-SVM	<	D-SVM	<	I-SVM	<*	I-ARIMA	<	D-ARIMA
		4	D-ARIMA-SVM	<	I-ARIMA-SVM	<	D-SVM	<	I-SVM	<*	I-ARIMA	<	D-ARIMA
		6	D-SVM	<	I-ARIMA-SVM	<	D-ARIMA-SVM	<*	I-SVM	<*	D-ARIMA	<	I-ARIMA
		8	D-SVM	<	D-ARIMA-SVM	<	I-ARIMA-SVM	<	I-SVM	<*	I-ARIMA	<	D-ARIMA
		12	I-SVM	<	D-ARIMA-SVM	<	D-SVM	<	I-ARIMA-SVM	<	D-ARIMA	<	I-ARIMA
		18	D-ARIMA-SVM	<	D-SVM	<	I-ARIMA-SVM	<*	I-SVM	<	D-ARIMA	<	I-ARIMA
		24	I-SVM	<	D-SVM	<	D-ARIMA-SVM	<	I-ARIMA-SVM	<	I-ARIMA	<	D-ARIMA
		1–24	D-ARIMA-SVM	<	D-SVM	<	I-ARIMA-SVM	<	I-SVM	<*	I-ARIMA	<	D-ARIMA

Note: * The mean difference between the two adjacent strategies is significant at the 0.05 level.

## References

[1] Cigliano J (1980). Price and income elasticities for airline travel: the North Atlantic market. *Business Economics*.

[2] Abed SY, Ba-Fail AO, Jasimuddin SM (2001). An econometric analysis of international air travel demand in Saudi Arabia. *Journal of Air Transport Management*.

[3] Grosche T, Rothlauf F, Heinzl A (2007). Gravity models for airline passenger volume estimation. *Journal of Air Transport Management*.

[4] Jorge-Calderón JD (1997). A demand model for scheduled airline services on international European routes. *Journal of Air Transport Management*.

[5] Russon MG (1990). Iterative nonlinear estimation of air passenger flow sensitivity to political boundaries and a complex function of distance. *Logistics & Transportation Review*.

[6] Grubb H, Mason A (2001). Long lead-time forecasting of UK air passengers by Holt-Winters methods with damped trend. *International Journal of Forecasting*.

[7] Donate JP, Cortez P, SáNchez GG, De Miguel AS (2013). Time series forecasting using a weighted cross-validation evolutionary artificial neural network ensemble. *Neurocomputing*.

[8] Lu C-J, Lee T-S, Chiu C-C (2009). Financial time series forecasting using independent component analysis and support vector regression. *Decision Support Systems*.

[9] Kao LJ, Chiu CC, Lu CJ, Yang JL (2013). Integration of nonlinear independent component analysis and support vector regression for stock price forecasting. *Neurocomputing*.

[10] Hu Z, Bao Y, Xiong T (2013). Electricity load forecasting using support vector regression with memetic algorithms. *The Scientific World Journal*.

[11] Xiong T, Bao Y, Hu Z (2013). Beyond one-step-ahead forecasting: evaluation of alternative multi-step-ahead forecasting models for crude oil prices. *Energy Economics*.

[12] Tatinati S, Veluvolu KC (2013). A hybrid approach for short-term forecasting of wind speed. *The Scientific World Journal*.

[13] Bao Y, Xiong T, Hu Z (2012). Forecasting air passenger traffic by support vector machines with ensemble empirical mode decomposition and slope-based method. *Discrete Dynamics in Nature and Society*.

[14] Alekseev KPG, Seixas JM (2009). A multivariate neural forecasting modeling for air transport—preprocessed by decomposition: a Brazilian application. *Journal of Air Transport Management*.

[15] Pai P-F, Lin C-S (2005). A hybrid ARIMA and support vector machines model in stock price forecasting. *Omega*.

[16] Guo Z, Zhao J, Zhang W, Wang J (2011). A corrected hybrid approach for wind speed prediction in Hexi Corridor of China. *Energy*.

[17] Chen K-Y, Wang C-H (2007). A hybrid SARIMA and support vector machines in forecasting the production values of the machinery industry in Taiwan. *Expert Systems with Applications*.

[18] Zhang PG (2003). Time series forecasting using a hybrid ARIMA and neural network model. *Neurocomputing*.

[19] Chevillon G (2007). Direct multi-step estimation and forecasting. *Journal of Economic Surveys*.

[20] Box GE, Jenkins GM, Reinsel GC (2013). *Time Series Analysis: Forecasting and Control*.

[21] Brockwell PJ, Davis RA (2009). *Time Series: Theory and Methods*.

[22] Cox DR (1961). Prediction by exponentially weighted moving averages and related methods. *Journal of the Royal Statistical Society B*.

[23] Bao Y, Xiong T, Hu Z (2013). PSO-MISMO modeling strategy for multistep-ahead time series prediction. *IEEE Transactions on Cybernetics*.

[24] Andrawis RR, Atiya AF, El-Shishiny H (2011). Forecast combinations of computational intelligence and linear models for the NN5 time series forecasting competition. *International Journal of Forecasting*.

[25] Önöz B, Bayazit M (2003). The power of statistical tests for trend detection. *Turkish Journal of Engineering and Environmental Sciences*.

[26] Sorjamaa A, Hao J, Reyhani N, Ji Y, Lendasse A (2007). Methodology for long-term prediction of time series. *Neurocomputing*.

[27] Sharma A (2000). Seasonal to interannual rainfall probabilistic forecasts for improved water supply management: part 1—a strategy for system predictor identification. *Journal of Hydrology*.

[28] Bao Y, Hu Z, Xiong T (2013). A PSO and pattern search based memetic algorithm for SVMs parameters optimization. *Neurocomputing*.

[29] Kennedy J, Eberhart R Particle swarm optimization.

[30] Hyndman RJ, Koehler AB (2006). Another look at measures of forecast accuracy. *International Journal of Forecasting*.

[31] Goodwin P, Lawton R (1999). On the asymmetry of the symmetric MAPE. *International Journal of Forecasting*.

[32] Qi M, Zhang GP (2008). Trend time-series modeling and forecasting with neural networks. *IEEE Transactions on Neural Networks*.

[33] Tukey's B (1953). *Multiple Comparisons*.

[34] Koning AJ, Franses PH, Hibon M, Stekler HO (2005). The M3 competition: statistical tests of the results. *International Journal of Forecasting*.

[35] Chang C-C, Lin C-J (2011). LIBSVM: a library for support vector machines. *ACM Transactions on Intelligent Systems and Technology*.

[36] Kennedy JF, Kennedy J, Eberhart RC (2001). *Swarm Intelligence*.

[37] Ramsay F, Schaefer D (1996). *The Statistical Sleuth*.

